# TGFBI Gene Mutation Analysis of Clinically Diagnosed Granular Corneal Dystrophy Patients Prior to PTK: A Pilot Study from Eastern China

**DOI:** 10.1038/s41598-017-00716-5

**Published:** 2017-04-04

**Authors:** Li Zeng, Jing Zhao, Yingjun Chen, Feng Zhao, Meiyan Li, Connie Chao-Shern, Tara Moore, John Marshall, Xingtao Zhou

**Affiliations:** 1 Eye and ENT Hospital of Fudan University, Myopia Key Laboratory of China Health Ministry, Shanghai, China; 2 Ulster University, Northern Ireland, UK; 3 University College London, London, UK

## Abstract

This study investigated the TGFBI gene mutation types in outpatients clinically diagnosed with granular corneal dystrophy (GCD) prior to phototherapeutic keratectomy (PTK), also calculated the mutation rate of subjects with normal corneas, but positive family history. Clinical GCD outpatients and consanguineous family members were enrolled in this study. Among total 42 subjects: 24 patients from 23 unrelated families had typical signs of GCD on corneas; 5 patients from 5 unrelated families had atypical signs; 13 subjects from 11 unrelated families had no corneal signs but positive family history. Using Avellino gene test kit, the TGFBI mutation detection was performed on DNA samples from all subjects. 36 subjects were detected to carry heterozygous TGFBI gene mutations. Among 24 clinical GCD patients, the proportion of R124H, R555Q, R124L, R555W and R124C were 37.5%, 16.7%, 25.0%, 20.8% and 0%, respectively, and 2 patients had been diagnosed with GCD according to the opacities thriving after LASIK (R124H) and PRK (R555W). The mutation rate of 13 subjects having no signs but positive family history was 69.2%. R124H mutation is the most prominent mutation type among GCD outpatients in Eastern China. It is recommended to conduct gene detection for patients with positive family history prior to refractive surgeries.

## Introduction

Traditionally, the diagnosis and classification of corneal dystrophies (CD) are mainly based on cornea signs in addition to clinical symptoms. GCD is a type of stromal dystrophy and it has 2 types (GCD type 1, GCD type 2) according to clinical manifestation. However, atypical corneal dystrophy is often difficult to diagnose and classify by observing opacities on corneas. It could be misdiagnosed before PTK treatment or overlooked prior to refractive surgeries due to lack of signs^1, 2^.

Transforming growth factor, beta-induced, 68 kDa (TGFBI, also known as BIGH3) gene mutations has been reported to be the essential cause of GCD^3^, furthermore R124H and R555W are mutation hotspots of TGFBI gene^4^. The R124H mutation of TGFBI is highly specific and has strong correlation with GCD type 2 (GCD2), also known as Avellino corneal dystrophy^5–7^. Identification of gene mutation establishes the new GCD classification, standardized by the International Committee for Classification of Corneal Dystrophies, IC3D^1, 8^. There are also reports regarding the rapid gene detection methods^9, 10^. They provide more efficient detection and more accurate classification for corneal dystrophies, especially when no signs have appeared.

For outpatients prior to refractive surgery or PTK, more attention should be paid to the relationship between preoperative corneal abnormalities and gene mutations. However, few reports have been published revealing gene mutation types of outpatients before treatment and patients who have not developed significant opacities on corneas. Thus, in this study we carried out gene tests on patients prior to PTK and their consanguineous family members. To the best of our knowledge, this is the first introduction of Avellino gene detection kit for genetic diagnosis of GCD in China. It is also a rather early display of TGFBI gene for GCD patients prior to PTK and an evaluation of patients who have no signs but GCD family background.

## Results

TGFBI gene test results and clinical information of 42 subjects are shown in Table 1.

**Table 1:** Summary of the Gene Test Results.

	Gene Test Results					
	R124H Positive	R555Q Positive	R124L Positive	R555W Positive	R124C Positive	Negative
Typical signs on corneas	9	4	6	5	0	0
Atypical signs on corneas	3	0	0	0	0	2
No corneal signs, but positive family history	2	3	1	1	2	4
Total	14	7	7	6	2	6

Results of 42 subjects are all shown in Table 1, and the clinical features of subjects are listed in the left columns.

Among the 42 subjects, 36 were tested positive for gene mutations and 6 were negative. All 36 subjects carried heterozygous mutations; among them, 14 were tested positive for R124H mutation, 7 for R555Q, 7 for R124L, 6 for R555W and 2 for R124C mutation. 24 of them had typical signs of GCD, 3 had atypical signs and 9 had no signs but positive family history, according to the medical record of our study. Of 6 subjects carrying no mutation, 4 had no signs but positive family history, 2 had atypical signs.

### The TGFBI mutations found in clinical GCD patients with typical opacities on corneas

The 24 patients clinically diagnosed with GCD from 23 unrelated families all carried TGFBI gene mutations, and the proportion of R124H, R555Q, R124L, R555W and R124C mutation were 37.5%, 16.7%, 25.0%, 20.8% and 0%, respectively. Among them, 2 patients suffering from opacities arising after LASIK and PRK surgeries were detected with R124H and R555W mutations respectively.

### The TGFBI mutations found in patients with atypical signs

Among 5 patients who had atypical signs on corneas, there were 3 subjects (60%) who were detected with TGFBI gene mutations, and all of them carried R124H mutation.

### The TGFBI mutations found in patients with no signs but positive family history of GCD

Among 13 subjects who had no signs on corneas but had positive family history of GCD, 9 subjects were tested with mutations, furthermore 2, 3, 1, 1 and 2 subjects were detected with R124H, R555Q, R124L, R555W and R124C mutation, respectively; 4 subjects were negative; the positive rate here was 69.2%.

## Discussion

The fast and accurate gene tests provide early detection and diagnosis of different types of GCD. Considering the correlation between GCD types and treatment options, and cases of post-LASIK GCD exacerbation, performing gene tests before refractive surgeries or PTK is critical^11–13^.

In our study, 24 patients clinically diagnosed with typical GCD are from eight provinces and autonomous regions in Eastern China. The testing kit covered 5 types of TGFBI mutations: R555W, R124H, R124L, R555Q and R124C. According to the IC3D, edition 2, these mutations caused granular corneal dystrophy, type 1, granular corneal dystrophy, type 2, Reis-Bücklers Corneal Dystrophy, Thiel-Behnke corneal dystrophy and classic lattice corneal dystrophy, respectively. The results showed that R124H mutation (GCD type 2) is the most prominent in these 24 outpatients, making up the greatest proportion of 37.5%. This result suggests that in the Eastern China region, GCD type 2 (Avellino Corneal Dystrophy) is the most prominent type of clinical GCD outpatients pre PTK. Neither Thiel-Behnke Corneal Dystrophy (TBCD) nor lattice corneal dystrophy type 1 (LCD1), which are related to R555Q mutation and R124C mutation respectively, is one of the GCD types. However, these 2 types of corneal dystrophies were found in our study by molecular methods. This suggests the uncertainty of the traditional clinical classification of corneal dystrophies, underlining the accuracy of genetic classification.

Prior to this study, another cohort study reported that GCD2, associated with the R124H mutation, and GCD1, with the R555W, were the two most common forms of corneal dystrophies of the Chinese population^14^. A South Korean study of seventy-one families with a total of three hundred eighty-seven subjects showed that eighty subjects from sixty-one families had R124H multination, which was the prominent mutation in South Korean population^15^. A study from Japan in 2001 showed that R124H mutation accounted for 72% of all cases of corneal dystrophies^16^. These results indicate that the R124H mutation is the leading cause for corneal dystrophy in the Asian regions. And our study, aiming at the Eastern Chinese population and focusing at the type of GCD, suggesting that R124H is responsible for a major part of GCD patients from Eastern China. However, the distribution of different types of corneal dystrophy is not consistent in various ethnic groups. A H626R mutation was found to account for the majority of corneal dystrophies in Vietnam^17^, while the H626R and R555W mutations are mostly seen in the Mexican population^18^. Considering the vast population in China, the exact distribution of different types GCD in China remains to be determined by a larger population study.

In particular, among 24 patients clinically diagnosed with GCD, one case of positive R555W mutation was detected 7 years after the PRK surgery, and corneal opacities occurred in another case after the LASIK surgery and the genetic test confirmed positive R124H mutation. There are a huge number of refractive surgeries in China and, in the past, there was no rapid genetic testing prior to surgeries. The prevalence of GCD in China is remarkable, which signifies the potential risk of a missed GCD diagnosis prior to refractive surgeries. It is unfortunate that those two positive cases in our study were not detected until after refractive surgeries. This suggests that refractive candidates, with or without typical GCD signs, need operators’ attention to see if these patients have the potential to have corneal dystrophies. Reports indicate that other ethnic groups have similar situation as in China, where the gene test is not included in the preoperative routine examinations^19–21^. Thus, we recommend carrying out gene tests on patients who have positive family history before refractive surgeries as far as possible.

We included 13 patients who had no signs but positive family history in this study. Among them, 9 subjects were positive for TGFBI gene mutation and 4 were negative. Although the test kit covered only 5 types of mutations, the family tree record and the gene results ensured that each of the 4 negative subjects had at least 1 family member tested to be positive by this kit. Considering the inheritance of corneal dystrophies^8^, consanguineous GCD patients should carry the same mutation. Consequently, we suppose that the 4 negative results were not due to the limitation of gene test kits and the 4 negative subjects carried no mutations. In this study, we also focused on the mutation rate of people with positive family history of GCD. The rate of 69.2% demonstrates that patients with GCD positive family history are highly probable to carry gene mutations. Furthermore, we recommend performing genetic tests on the relatives of GCD patients. Early detection would provide timely observation and guidance for refractive surgeries. Patients in this study were all satisfied with the efficiency of the genetic tests, as the cotton-swab sampling method was convenient with a conclusion drawn in 48 hours. Nevertheless, alternative methods can be used for genetic analysis other than the test kit we used. It would be helpful to compare among different methods in terms of specificity and sensitivity in screening for potential mutation carriers before refractive surgery.

We also included 5 patients with atypical signs on corneas in our study. 3 patients were positive for R124H mutation, and 2 patients were tested negative. Before gene tests, these atypical corneal opacities made it difficult to diagnose and classify. The gene tests helped to confirm that 3 of them are GCD patients and avoid over-diagnosis of the other two. However, the gene test kit only covered 5 types of mutations and the 2 negative patients had no family history of GCD, suggesting that they could not be judged as “No gene mutation”, because the opacities on the corneas of these subjects could have been caused by other gene mutations. Nevertheless, only 2 out of 42 subjects exhibited suspicious results, indicating that the efficiency of this gene test kit was acceptable.

There are limitations for this study. First, only 5 types of mutations were covered by the gene test kit. As for the 2 subjects with atypical signs who were tested negative for mutation, we could not confirm that they carry no mutations. Gene sequencing of all exons of the TGFBI gene should help to achieve accurate diagnosis. Besides, all 24 clinical patients in our study were preoperative outpatients from Eastern regions of China. And the sample size is rather small. More information remains to be revealed by studies with larger sample sizes.

## Methods

Enrolled between Dec 1st, 2014 and Feb 15^th^, 2016, the pre-PTK outpatients clinically diagnosed with GCD, along with their consanguineous family members, formed a 42-subject group for this study. The significance and details of this study were introduced to the patients, and informed consent was obtained from all subjects. This study was approved by the Ethics Committee of Eye and ENT Hospital, Fudan University (Trial number 2015018). All examinations were performed in accordance with the Declaration of Helsinki.

### Patients

**Figure 1:**
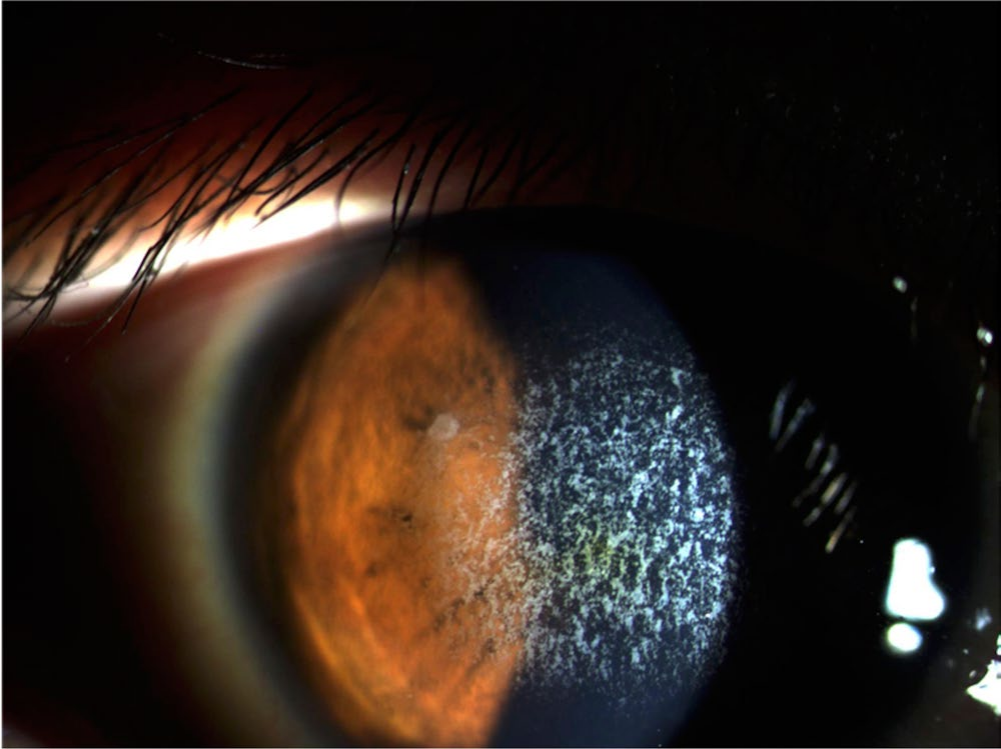
Slit-lamp photography of a patient who was clinically diagnosed with GCD. There were central granular corneal opacities on the cornea.

**Figure 2:**
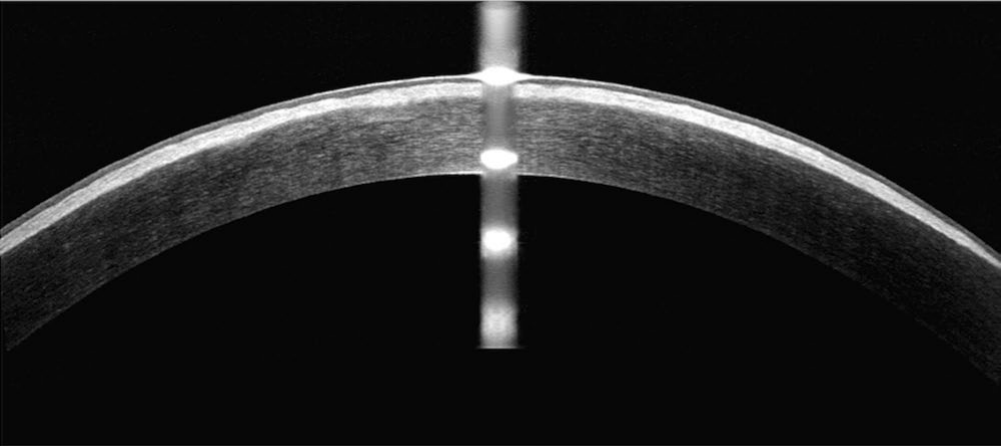
OCT findings in a patient with typical signs on the cornea. Significant opacities could be seen on the cornea.

**Figure 3:**
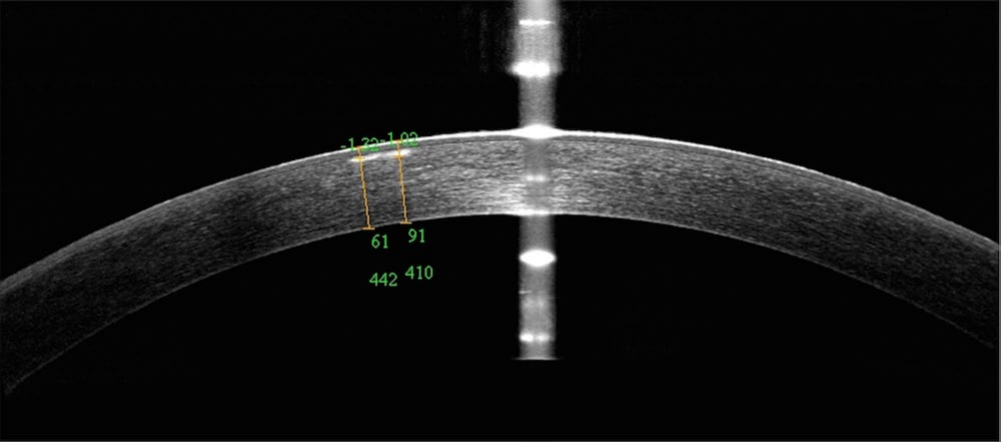
OCT findings in a patient with atypical signs. The opacity was small and atypical.

All the 42 subjects participated in the entire cohort. There were 23 males and 19 females ranging from 8 to 89 years of age. Information of patients’ symptoms, family history and previous surgical history were obtained. A family tree was constructed according to the information acquired. In addition, the slit lamp microscope examination and optical coherence tomography (OCT) images were recorded. We defined typical signs of GCD as diffusedly cluster, granular or crumb opacities on the corneas under the slit lamp observation (Fig. 1) and OCT (Fig. 2); atypical signs of GCD were observation of 1~2 sporadic or isolated scattering points on corneas (Fig. 3). According to the foregoing definitions, we can tell that the clinical situations of 42 subjects were as follows: (1) 24 patients with typical GCD signs were from 23 unrelated families. Among them 2 patients were diagnosed with GCD according to the opacities thriving after LASIK and PRK; (2) 5 patients from 5 unrelated families had atypical signs on corneas; (3) 13 subjects from 11 unrelated families had no symptoms or signs on corneas but they had positive family history of GCD, and each of them was a consanguineous family member of the typical GCD patient mentioned above, except for 3 subjects from 1 family, that the GCD patient in this family did not participate in our study.

### Gene Tests

All the 42 subjects participated in the whole cohort study and their DNA samples were tested with Avellino gene test kit, which had not been put into commercial use in China. This gene test kit covered five types of TGFBI mutations: R555W, R124H, R124L, R555Q and R124C. According to the IC3D, edition 2, these mutations caused Granular corneal dystrophy, type 1, Granular corneal dystrophy, type 2, Reis-Bücklers Corneal Dystrophy, Thiel-Behnke corneal dystrophy and classic Lattice corneal dystrophy respectively, constituting “TGFBI corneal dystrophy”. Epithelial cells were taken from subjects’ buccal mucosa with the buccal swabs, which was subsequently inserted into the protective outer tube. Testing laboratory performed DNA extraction by DNA Extract All Reagents (Life Technologies, 850 Lincoln Centre Drive, Foster City, CA 94404, USA) and DNA amplification by TaqMan GTXpress Master Mix (Life Technologies, 850 Lincoln Centre Drive, Foster City, CA 94404, USA). SPSS software 22.0 (IBM Corp., New York, NY, USA) was applied to analysis the test results.
